# 

**Published:** 1894-06-23

**Authors:** 


					No. 404. Vol. XVI.
Saturday,
June 23rd, 1894.
WITH EXTRA NURSING SUPPLEMENT. ??ce twopence.
[Registered at the General Post Office as a Newspaper for P?^/re-
transmission in the United Kingdom and Abroad.] a A' JP? 're0? 8d-
J ""to Per Annnm, 8s.6d.. post free.

				

